# Enhancement Effect of Shark Cartilage Extract on Treatment of *Leishmania infantum* with Artemisinin and Glucantime and Evaluation of killing Factors and Apoptosis *in-vitro* Condition

**DOI:** 10.22037/ijpr.2019.1100656

**Published:** 2019

**Authors:** Soheila Molaie, Fatemeh Ghaffarifar, Zuheir Mohammad Hasan, Abdolhosein Dalimi

**Affiliations:** a *Department of Parasitology , Faculty of Medical Sciences, Tarbiat Modares University, Tehran, I.R.Iran.*; b *Department of Immunology, Faculty of Medical Sciences, Tarbiat Modares University, Tehran, I.R.Iran.*

**Keywords:** Artemisinin, Glucantime, Shark cartilage extract, Apoptosis, In-vitro, Leishmania infantum

## Abstract

In this study we examined enhancement effects of Artemisinin plus Glucantime and shark cartilage extract on promastigotes and amastigotes of *L.infantum *in *in-vitro* condition.The toxicity of artemisinin, glucantime, and shark cartilage extract on the *L. infantum *promastigotes and amastigote-infected macrophages was evaluated using MTT assay. The role of these drugs inducing apoptosis in promastigotes, un- infected, and parasite- infected macrophages was also studied. Using promastigote assay, IC50 values of artemisinin and glucantime as standalone drugs as well as in combination were obtained to be 50, 400, and 100µg/mL respectively. The flow cytometry analysis of apoptotic promastigotes stained with Annexin-V FITC staining showed that artemisinin, glucantime, artemisinin plus glucantime, artemisinin plus shark cartilage extract, and shark cartilage extract alone applied at their IC50 concentrations resulted in 53.5%, 73.92%, 64.46%, 49.9%, and 47.34% apoptosis respectively. The results of MTT assay indicated that cytotoxicity of artemisinin, glucantime, artemisinin plus glucantime, shark cartilage plus artemisinin, and shark cartilage in infected macrophages after 72h was 75%, 84%, 82%, 30%, and 3% respectively. In un- infected macrophages, cytotoxicity of Artemisinin, Glucantime, Artemisinin plus Glucantime and shark cartilage was 15%, 31%, 21%, 2%, and 0% respectively.This study suggests that artemisinin, glucantime, artemisinin plus glucantime, and shark cartilage extract have significant killing effects on promastigotes and amastigotes. Also, it proved that artimisinin alone and in combination with glucantime and shark cartilage extract has little toxic effect on macrophages, but could induce apoptosis in *L.infantum *promastigotes and amastigote-infected macrophages. Thus, these chemicals can be used as alternative drugs for *in-vivo* studies.

## Introduction

Leishmaniasis is one of the globally neglected tropical diseases that threaten almost 350 million people of all ages in 98 countries. Reports indicate that the prevalence of the disease is 12 million with a mortality rate of 60,000 cases per year (1). A recent survey showed that 0.2 to 0.4 million new visceral leishmaniasis cases and 0.7 to 1.2 million cutaneous leishmaniasis cases occur each year (2). Leishmaniasis is caused by a protozoan parasite that proliferates in certain vertebrates as reservoirs. The disease transmit to human with bites of infected phlebotomine females. The development of the disease depends on *Leishmania* species, conditions of the patient immunity, and rate of immune response .Visceral leishmaniasis is a serious form of the disease which results in epidemics of high fatality rate in endemic areas, if left untreated (3). In Iran, visceral leishmaniasis mostly infects children under 2 years of age (4).

In the absence of effective vaccine, the disease control is mainly based on chemotherapy. Drugs such as pentavalant antimonials (5), paromomycin deoxycholate (6), sitamaquine (7), amphotericin B (8), and miltefosine (9) are currently using for visceral leishmaniasis treatment, but these drugs are expensive, teratogenic and have adverse side effects and long half-time (9). Thus, less toxic, unexpensive and less persistant drugs are indeed necessary.

 In recent years, some of important herbal medicines were used as anti-leishmaniasis. Plant componds including Alkaloids, Flavonoids, Saponins, Quinones, and Chalcones were used in leishmaniasis control (10, 11). Also, trepenoids including monoterpenes and sesquiterpens were introduced as leishmaniacidal compounds. Artemisinin (Art) (or *qinghaosu *in Chinese), a sesquiterpene trioxane lactone produced solely in glandular trichomes (GLTs) of *Artemisia annua* Leaves. It is currently the best treatment of malaria (12). Artemisinin is a traditional chinese medicine which owes its biological activity related to an endoperoxide bridge in its chemical structure (13, 14). Artemisinin and its derivatives exhibit a quick onset of action but leave the blood rapidly. They generate bioreactive radicals capable of intracellular damages (15). Some studied showed that artemisinin and its derivatives have therapeutic potential against malaria (15) and various degree of effectiveness on infections caused by *Leishmania* species (16, 17), *Schistosoma* (18), chlonorchis (19), *Fasiola* (20), *Toxoplasma* (21), viruses (22, 23), bacteria (24), and fung (25). Artemisinin has also shown promissing anticancer effects (26). 

On the other hand, some studies showed that the leishmania treatment relies on modulation of patient immune response (27). In recent years, shark cartilage was considered as immunomodulator (28).

Shark cartilage is acquired from freshly caught spiny dogfish sharks and hammerhead sharks in the Pacific Ocean (29). Shark cartilage has constitute several chemical materials such as Proteins ( troponin-I, tetranectin-type protein, collagenases, cartilage-derived inhibitor (CDI), tissue inhibitors of metalloproteinases (TIMPs), Glycoproteins(shyrnastatin-1 and -2, galactosamine, glucosamine), and Glycosaminoglycans ( chondroitin sulfate-D, chondroitin-6-sulfate, keratan sulfate) ( 30). Shark cartilage has been used for thousands of years in China as a health product called shark fin soup. In different studies, researchers showed several property of shark cartilage : angiogenesis inhibitor in the treatment of cancer (31), as a joint lubricant in arthritis (32), treat psoriasis and diabetic retinopathy (33) and as immunomodulator: Induces Th1type inflammatory cytokines (34).

Shark cartilage is composed of two peptides, with molecular mass of 14 and 15 kD, which preferentially induces Th_1_ type cytokines such as IFN-γ (28).

The main cells involved in cellular defense against *Leishmania* infection are macrophages. During infection, the parasite enters the macrophage via interference of surface receptors and converts to amastigotes (27).

 In this study, we evaluated the effect of Artemisinin, as stand-alone or in combination with glucantime (Glu) as current drug in leishmania treatment and shark cartilage extract (SCE), on *Leishmania infantum* promastigotes, as well as on both infected and non-infected macrophages. We also studied the cytotoxic effect of these compounds on the parasite apoptosis. 

## Experimental

*Material and methods*

*Ethics Statement*

This project was approved by Ethical Committee on 27^th^ April 2015, School of Medical Sciences Tarbiat Modarres University (adopted from the Declaration of Helsinki (1975) and the Society for Neuroscience Animal Care and Use Guidelines (1998). 

*Parasite and Drug preparation*

For this experimental study, *Leishmania infantum* (MCAN/ES198/LIM-877) was obtained from Medical Science of Kerman University. Promastigotes were cultured in RPMI 1640 (Gibco,US) enriched with FBS 20% (Fetal Bovine Serum) (Gibco, US), 100 IU/mL of penicillin G and 100µg/mL of streptomycin and then incubated in 18-24 °c.

Artemisinin(C15H22O5) (Mw:282.4) was purchased from Holly pharmaceuticals (US) company. Stock solutions of the drug were freshly prepared with 1/1 ratio of ethanol and distilled water (35). Glucantime was purchased from Aventis company (France) and shark cartilage was obtained from Bandar Bushehr city (Southern Iran) by Prof. Zuheir M Hassan.

*Preparation of shark cartilage extract (SCE)*

At first, shark cartilage was cleaned carefully and washed with distilled water. The shark cartilage extract was prepared according to a method described by Feyzi and *et al* (28). Brifly, the cleaned cartilage was cut into small pieces, lyophilized and then pulverized. Ten grams of the cartilage powder was extracted in 100 mL of 0.1M citrate buffer containing 4 M guanidine HCl and a protease inhibitor cocktail (EDTA 6.25 mM, PMSF 1 mM) at pH = 5.8 for 48 h with slight stirring at 2-8 ºC. The extract was then centrifuged at 100,000 g for 45 min. The supernatant was dialyzed against PBS (15). 

*Promastigote assay*

In experimental group, *L. infantum* promastigotes were exposed to different concentrations of Artemisinin, Glucantime, and shark cartilage extract as standalone drugs or to Artemisinin in combination with either of the other two drugs. In negative control group, promastigotes were cultured as triplicate without addition of any drug. We also used Amphotricin B as a positive control.

To obtain 50% inhibitory concentration (IC50) of drugs‌‌‌‌(Artemisinin, Glucantime, Artemisinin+Glucantime) on *leishmania infantum* promastigotes, microtitration plate (96 well) was used. First, 100 µL of 10^6 ^/mL promastigotes (20) in logarithmic phase were added to each well. Then, 100 µL of different concentration (3.12-400 µg/mL of Artemisinin, Glucantime, and Artemisinin plus Glucantime), 100 and 50 µg/mL of Artemisinin plus shark cartilage extract and shark cartilage extract alone were added to wells numbered 1 to 8 (each one was in triplicate). Then, the plate was incubated at 24 °C and the number of promastigotes was counted after 24,48, and 72 h using a neubar chamber. 

The IC50 values of drugs were then determined based on the results of 72 h count by drawing a specified chart. 

*Colorimetric MTT assay for detection of cell viability*

The anti-leishmanial activity of Artemisinin, Glucantime, Artemisinin plus Glucantime, shark cartilage extract, and Artemisinin plus SCE against promastigotes was measured by the MTT assay. Briefly, log phase promastigotes (1×10^6^ cells/200 μL/well) were incubated with Artemisinin, Glucantim, Artemisinin in combination with Glucantime (3.12 – 400 µg/mL concentrations), and shark cartilage extract (100, 50, 25 µg/mL concentrations) for 72 h at 24 °C. At the end of 72 h, a solution comprising MTT(5 mg/mL distilled water) was added at 20 μL per well. The plates were then incubated for further 5 h at 37 °C in a dark room. The cells were centrifuged at 3000 rpm for 10 min and 100 μL DMSO (dimethyl sulfoxide) was added to pellets and then incubated again. After 10 min, optical density (OD) of plate was read by an ELISA reader at 570nm.Viability percentage was calculated using the following formula: 100× (absorbance of treated cells/absorbance of control cells).

*Amastigote assay*

*Macrophage culture*

For the purpose of this study, J774 macrophages were obtained from the stock of Tarbiat Modarres University, Department of Medical Parasitology, stored at -70 °C. At first J774 were cultured in RPMI 1640 with FBS 10%, 100 IU /mL penicillin G and 100µg/mL streptomycin, then incubated at 37 °C in 5% CO2 atmosphere. 

*Cytotoxic assay by MTT*

 The toxicity of target drugs (Artemisinin,Glucantime,Artemisinin+Glucantime, shark cartilage extract and shark cartilage extract plus Artemisinin) on non-infected and infected macrophage cells were evaluated using MTT test. Using 96-Well microplates (Nunc), 100 µLof the macrophage cultures (1 × 10^5^ cell/mL) were plated in wells. After the cells had bonded to bottom of plate, the culture medium was removed and replaced with fresh culture medium (100 µg/mL ) comprising different concentration of target drugs (3.12–400 µg /mL ). After 72 h of culture, the viability of the macrophages was measured. 

Infected macrophages was prepared by adding promastigotes of *L. infantum* in the stationary phase to macrophage cultures in wells at a ratio of 10 parasites per macrophage. The plate was incubated in a CO2 incubator (37 ºC, 5% CO2, and 95% humidity) for 24 h, to the infected macrophage cells by the promastigotes. Extra free parasites were then washed, and the cells were incubated for 24 h in culture medium alone. This medium was thrown away and the cells were incubated at 37 ºC for 72 h in fresh medium that posses different concentrations (3.12–400 µg/mL) of drugs. After 72 h of culture, the viability of the macrophages was measured. Uninfected macrophages, and *L .infantum-*infected macrophages without any drugs were used as control cells.

*Amastigote inhibition test (infectivity assay)*

J774 macrophage Cells were seeded at a density of 1×10^5^ cells/well in 24-well microplates (Nunc) with rounded coverslips in the bottom and cultured for 24 h. The cells were then infected *in-vitro* with promastigote forms of *L. infantum* at stationary phase at a ratio of 10:1. After 6 h incubation, non-phagocytosed parasites were removed by washing. Infected macrophages were further incubated in the presence or absence (as negative control group) of Artemisinin (50, 100 µg/mL), Glucantime (200, 400 µg/mL), Artemisinin+Glucantime (Art 50 µg/mL+Glu 400 µg/mL, Art 100 µg/mL+Glu 400 µg/mL), Artemisinin 50 and 100 µg/mL plus shark cartilage extract and shark cartilage extract alone for 72 h. Drug activity was determined on the basis of both the percentage of infected cells and the number of amastigotes per infected cell in treated and untreated wells in methanol-fixed and Giemsa-stained preparation.Values are the means of three separate experimentations.

*Flowcytometry assay*

The Annexin-V FLUOS Staining Kit (Bio-vision, USA) was used for detecting apoptotic and necrotic cells. The promastigotes were cultured in 24 well plates (1 × 10^6^ parasites/well) in the absence (as negative control group) and the presence of Artemisinin at 100, 50, 25 µg/mL concentrations, Glucantime at 200, 400 µg/mL, combination of both drugs, combination of Artemisinin plus shark cartilage extract and shark cartilage extract alone.

**Figure 1:**
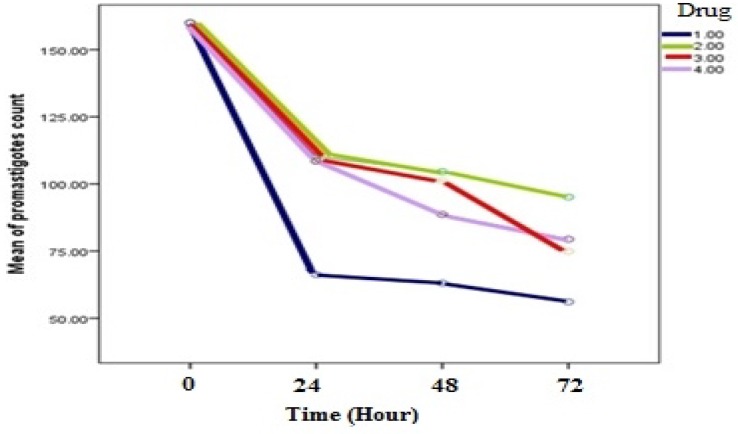
Comparision of the effect of four drugs on *L. infantum *promastigotes count: Artemisinin(1), Glucantime (2), Comination of Artemisinin with Glucantime (3) and shark cartilage extract (4) at time intervals of 24, 48 and 72 h (*p *value < 0.001)

**Figure 2:**
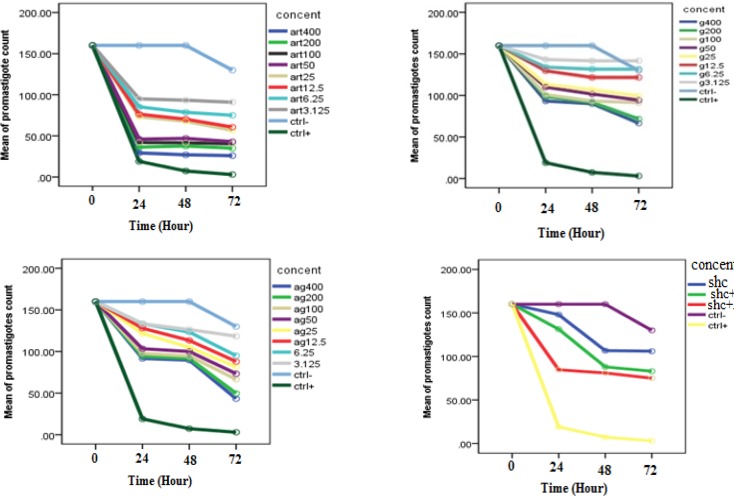
The mean of *L. infantum *Promastigotes count in Presence of different Concentrations of Artemisinin(art), Glucantim(g), Artemisinin plus Glucantim(ag) and Shark cartilage extract, Shark cartilage extract (shc) plus Artemisinin (Art) in comparison with control group at different time intervals of 0, 24, 48 and 72 h (*p *< 0.001)

**Figure 3:**
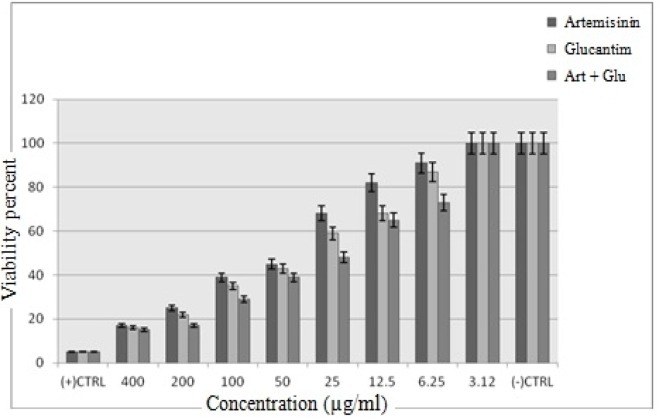
The viability percentage of promastigotes following treatment with various concentrations of Artemisinin, Glucantime, and Art plus Glu (*p *< 0.001)

**Figure 4:**
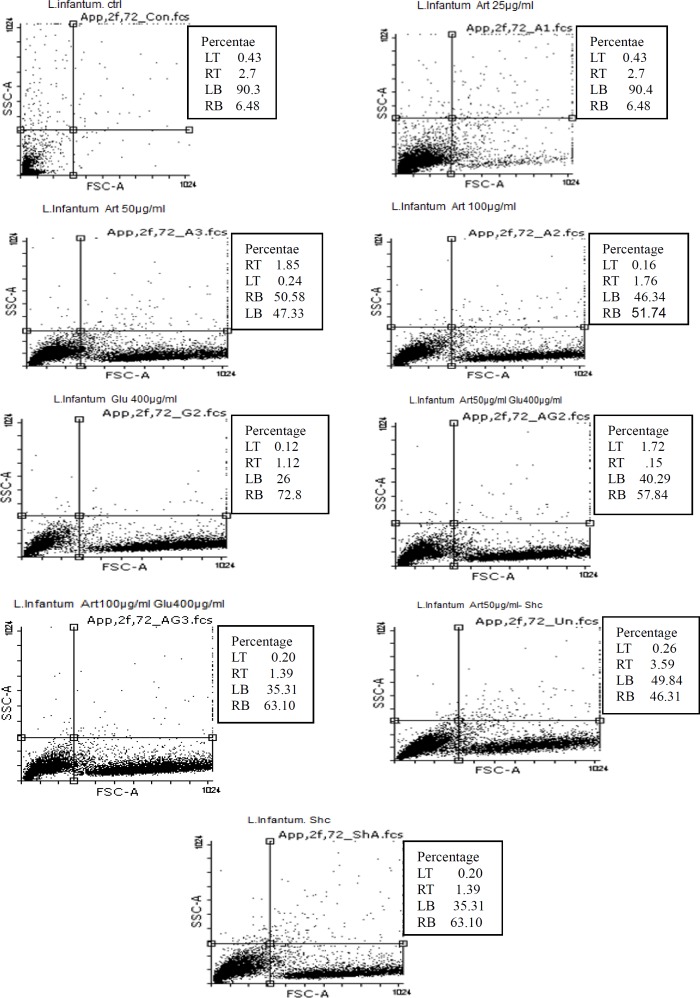
Flow cytometry results. Promastigotes staining with Annexin V and Propidium Iodide after treatment with different concentrations of target drugs after 72 h

**Figure 5:**
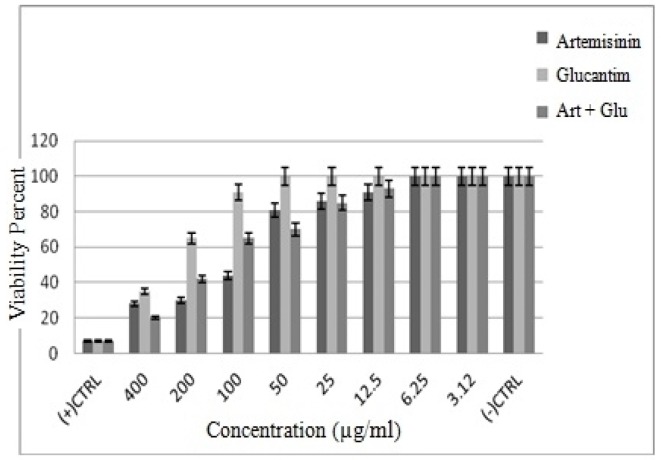
The viability of infected macrophage cells at various concentrations of Artemisinin, Glucantime and Artemisinin (Art) plus Glucantim (Glu) (*p *= 0.045)

**Figure 6:**
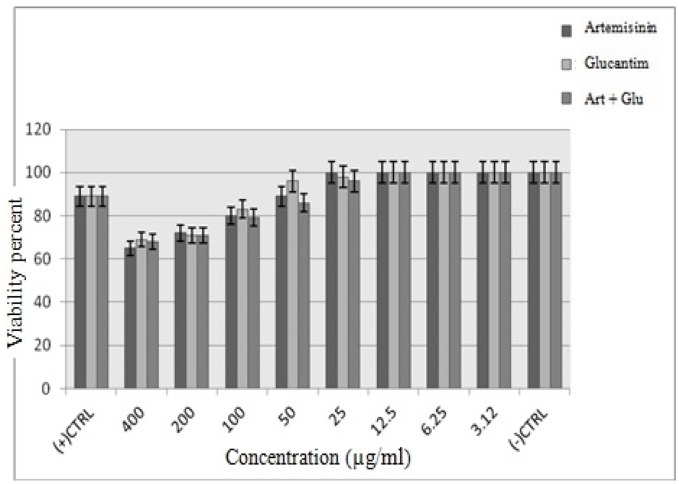
The viability of non-infected macrophage cells at various concentrations of Artemisinin, Glucantime and Artemisinin (Art) plus Glucantim (Glu) (*p *< 0.05)

**Figure 7:**
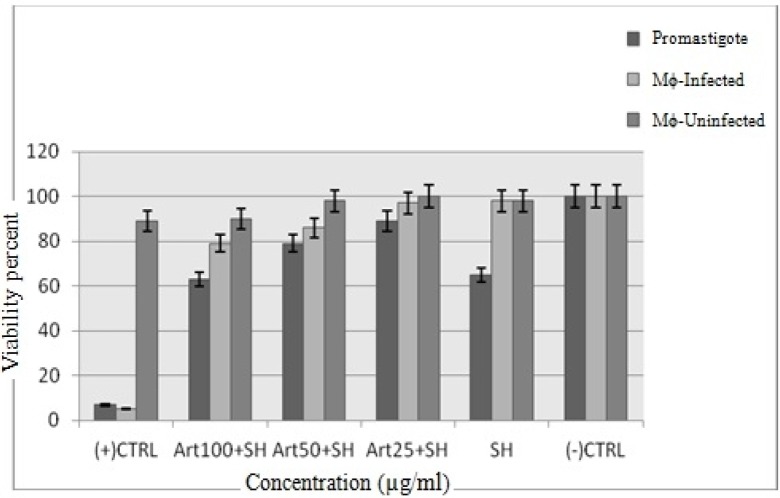
The viability percent of *L. Infantum *promastigotes, non-infected and infected macrophage cells with *L. infantum *promastigotes at shark cartilage extract and three concentrations of Artemisinin (25, 50 and 100 µg/mL) plus shark cartilage extract (*p *< 0.05)

**Figure 8:**
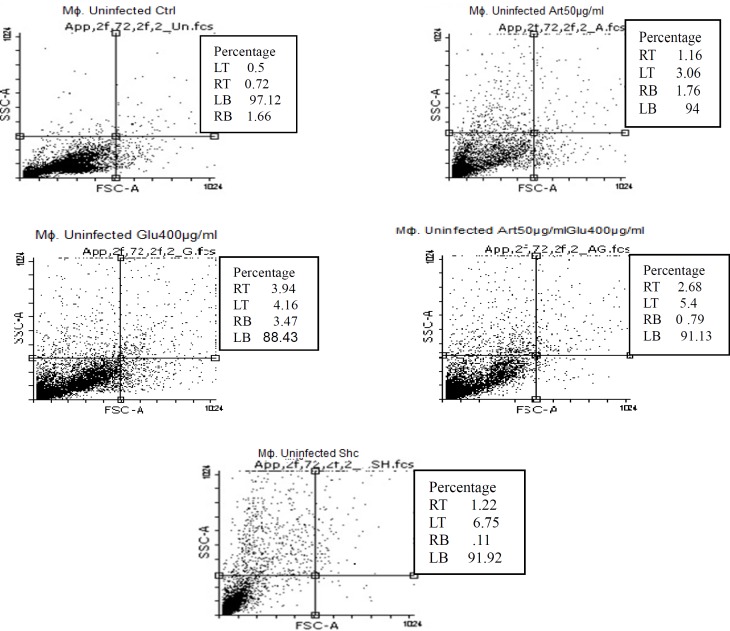
Flow cytometry results. Uninfected macrophage cells staining with Annexin V and Propidium Iodide after treatment with different concentrations of target drugs after 72 h.

**Figure 9:**
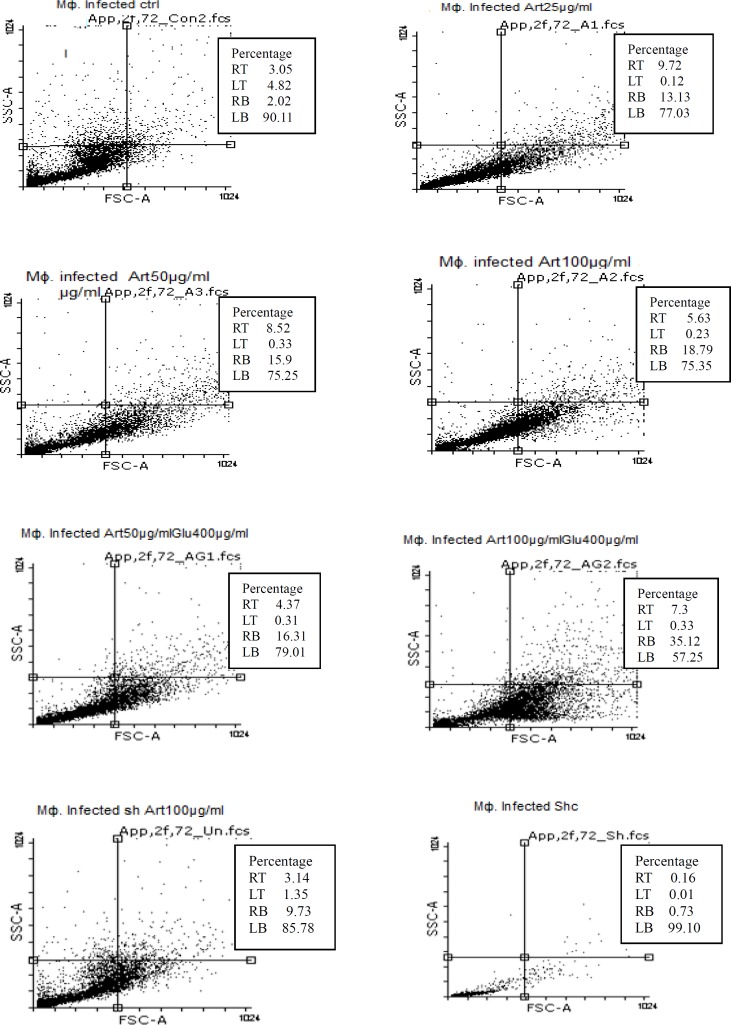
Flow cytometry results. Infected macrophage cells staining with Annexin V and Propidium Iodide after treatment with different concentrations of target drugs after 72 h.

**Table 1:** The effects of different concentrations of Artemisinin, Glucantime, Artemisinin+Glucantime, Shark cartilage extract and Artemisinin plus Shark cartilage extract on the number of promastigotes of L. *infantum (*×*106)*. Data are expressed as the mean ± SD (n = 3)

**Drug**	**24h**					**48h**					**72h**				
**concentration**	**Art**	**Glu**	**Art+Glu**	**SCE+Art**	**SCE**	**Art**	**Glu**	**Art+Glu**		**SCE**	**Art**	**Glu**	**Art+Glu**	**SCE+Art**	**SCE**
400	0.26±0.02	0.9±0.02	0.9±0.02	-	-	0.27±0.02	0.9±0.05	0.8±0.05	-	-	0.26±0.03	0.6±0.07	0.4±0.02	-	-
200	0.36±0.02	0.9±0.01	0.9±0.04	--	-	0.37±0.05	0.9±0.02	0.9±0.02	-	-	0.35±0.05	0.7±0.1	0.5±0.05	-	-
100	0.42±0.02	0.9±0.07	0.9±0.02	0.8±0.03	-	0.41±0.01	0.9±0.02	0.9±0.04	0.7±0.01	-	0.40±0.01	0.9±0.03	0.6±0.02	0.7±0.1	-
50	0.46±0.03	1±0.01	0.1±0.05	1.3±0.02	-	0.47±0.02	1±0.07	1±0.01	0.5±0.02	-	0.43±0.02	0.9±0.04	0.7±0.02	0.8±0.03	-
25	0.73±0.02	1±0.05	1.2±0.02	-	-	0.67±0.08	1±0.05	1±0.08	-	-	0.56±0.02	0.9±0.1	0.8±0.02	-	-
12.5	0.75±0.02	1.2±0.05	1.2±0.02	-	-	0.70±0.1	1.2±0.1	1.1±0.1	-	-	0.60±0.04	1.2±0.02	0.8±0.02	-	-
6.25	0.85±0.03	1.3±0.03	1.3±0.02	-	-	0.73±0.03	1.3±0.02	1.2±0.02	-	-	0.75±0.05	1.3±0.02	0.9±0.05	-	-
3.12	0.95±.05	1.4±0.02	1.3±0.02	-	-	0.93±0.2	1.4±0.02	1.2±0.05	-	-	0.91±0.03	1.4±0.02	1.1±0.07	-	-
Control (-)	1.6±0.17	1.6±0.17	1.6±0.17	1.6±0.17	1.1±0.03	1.6±0.17	1.6±0.17	1.6±0.17	1.6±0.17	0.8±0.03	1.3±1.7	1.3±1.7	1.3±0.17	1.3±1.7	0.7±0.00
Control (+)	0.19±0.36	0.19±0.36	0.19±0.03	0.19±0.36		0.73±0.2	0.73±0.2	0.07±0.02	0.73±0.2		0.03±0.17	0.03±0.17	0.03±0.01	0.03±0.01	

**Table 2:** The percentage of infected macrophage cells and intracellular amastigotes at 72 h after treatment with different concentration of drugs ( *p *< 0.05)

Drug	Percentage of infected macrophages*	Percentage of intracellular amastigotes*
Artemisinin 25	60	42
Artemisinin50	55	38
Artemisinin100	50	35
Glucantime200	47	29
Glucantime400	41	23
Arte50+Glu400	40	20
Art100+Glu400	35	18
Art100+Sh	49	35
Sh extract	61	44
Ctrl (-)	63	45

* Data are expressed as the triplicate examines

The plates were then incubated at 24 °C. Following the kit instructions, the promastigotes were collected after 72h incubation and centrifuged at 3000 rpm for 5 min. Then the supernatant was discharged, and 500μL binding buffer, 5μL annexin V and 5μL propidium iodide (PI) were added to the residue. The samples were incubated at room temperature and dark situation for 5 min. The cell death were obtained by FACS Canto and were analyzed by FlowJo software.

To detect apoptosis of macrophage cells, 100µL medium culture containing 10^5^cells/mL was asded to wells. The wells were then treated with Artemisinin, Glucantime, Artemisinin plus Glucantime, and Artemisinin plus shark cartilage extract. To collect macrophages, 0.1% trypsin with 0.1% EDTA were used. Flow cytometry analysis was used to assess infected and non-infected macrophage cells.

*Statistical analysis*

At first, we tested sphericity hypothesis to validate four drug results. The repeated measure Anova were then used to analyse the obtained results with SPSS version 21(at *p* <0.05). The of date MTT tests were subjected to Shapiro-Wilk analysis of normality tests before being evaluated by Levene test for homogeneity of variances. In case of violtion of homogeneity of variances, the results were analyzed by Brown-Forsythe test which is a robust test of equality of means (*p* <0.005). 

## Results

*The results of promastigote assay*

*Inhibitory effects against promastigote forms*

Evaluation of inhibitory effects of various concentrations of Artemisinin*, *Glucantime and also combination of these two drugs and shark cartilage extract against promastigote of *L.infantum*, were done after 24, 48, and 72 h. The results showed that the growth inhibitory effect is dose and time dependent. Whereas, Artemisinin was the most active drug, its combination with Glucantime and shark cartilage extract showed to be more potent anti- promastigote than Glucantime alone. Moreover, the IC50 values of Artemisinin, Glucantime and Artemisinin plus Glucantime against promastigote forms of *L.infantum *were 50 µg/mL, 400 µg/mL, and 100 µg/mL respectively (Figure1, 2) (Table1).

*Viability of promastigote*

The cytotoxic effect of Artemisinin, Glucantime and Artemisinin with Glucantime combination on promastigotes of *L*. *infantum *was evaluated at eight concentrations (3.12 -400 μg/mL) after 72 h. The concentration of 400 and 3.12 μg/mL of these drugs after 72 h showed maximum and minimum cytotoxic effect on promastigotes of *L*. *infantum*. 

The results showed that by the drug concentration increase, the viability of promastigotes decreases. The viability of promasrigotes at the highest concentration (400 µg/mL) of Artemisinin,Glucantime, Artemisinin+Glucantime drugs, were 28%, 35% and 20% respectively. whereas that in the presence of Artemisinin,Glucantime and Artemisinin plus Glucantime at IC50 concentrations were 44%, 35%, and 91% respectively (Figure 3).

*Induced apoptosis by target drugs in promastigotes of L. infantum*

Flow cytometric analysis showed that necrotic and apoptotic effects in the parasite occur following treatment of promastigotes with three concentrations (25 , 50 and 100 μg/mL) of Artemisinin after 72 h. The percent of apoptosis (Early and Late Apoptosis) in promastigotes induced by 25, 50, and 100 μg/mL of Artemisinin after 72 h, was 9.18%, 52.43%, and 53.5% respectively. Induced apoptosis (Early and Late Apoptosis) in promastigotes induced by 200 and 400 μg/mL of Glucantime after 72 h were 71.65% and 74%, respectively, whereas those induced by 50, 100 μg/mL of Artemisinin plus 200μg/mL of Glucantime were 59.5% and 64.5% respectively. 

The percentages of apoptosis (Early and Late Apoptosis) in promastigotes induced by 50μg/mL of Artemisinin plus shark cartilage extract and shark cartilage extract alone after 72 h were 49.9% and 47.34% respectively (Figure 4).

*The results of Amastigote assay*

*Viability of J774 macrophage cells*

Similar to promastigote stage, the anti-amastigotes effects of various concentrations of Artemisinin, Glucantime and also Artemisinin plus Glucantime were dose-dependent response. Artemisinin as the main drug of the present study was the most active against intra-macrophage amastigotes of *L. infantum*. The cytotoxic effect of drugs on J774 macrophage cells was evaluated at 8 concentrations namely 400, 200,100,50,25,12.5,6.25 and 3.12 μg/mL after 72h. At IC50 concentrations of Artemisinin, Glucantime and Artemisinin plus Glucantime the viability of infected macrophage cells were 20%, 16% and 20%, respectively, whereas the viability of non-infected macrophage cells under the same drugs were 80%, 69%, and 79% respectively (Figures 5, 6).

*The results of shark cartilage extract at MTT assay*

The cytotoxic effect of shark cartilage extract alone and Artemisinin plus shark cartilage extract on promastigotes of *L*. *infantum, *non- infected and infected macrophage cells was evaluated at three concentrations (25, 500 and 100 μg/mL) after 72 h. The viability percent at the presence of shark cartilage extract on promastigotes, un- infected and infected macrophage cells were 63%, 98% and 98%, respectively, whereas at the presence of Artemisinin 50 µg/mL (IC50 of drug) plus shark cartilage extract were 78%, 98% and 85% respectively. So results showed that Shark cartilage extract did not have noticieable toxicity on infected and non- infected macrophage cells but had remarkable toxicity on promastigotes (Figure 7).

*Anti-amastigote assay*

The anti-amastigote activities of target drugs were evaluated in infected macrophage. The wresults showed that by increasing the drugs concentration, the number of amastigotes in infected macrophages decreases.The results also indicated that the percentage of infected macrophages and intracellular amastigotes decrease. Mean number of amastigotes /macrophage after 72 h in the negative control group was 45 while in Artemisinin group ( at 25, 50 and 100 μg/mL), Glucantime group (at 200 and 400 μg/mL), Artemisinin (50 µg/mL) plus Glu (400 µg/mL), Art (100 μg/mL) plus Glu (400 μg/mL), Artemisinin (100 μg/mL) plus shark cartilage extract and shark cartilage extract were 42%, 38%, 35%, 29%, 23%, 20%, 18%, 35% and 44% respectively (**Table 3**). These results showed a significant reduction of percentage of amastigotes in test groups compared to the control groups. On the other hand, the combination of artemisinin plus glucantime were much more effective than artemisinin or glucantime alone (Table 2).

*Drug Induced Apoptosis in L. infantum infected and non-infected macrophage cells*

 Flow cytometric analysis showed that necrotic and apoptotic effects on the parasite occur following treatment of non-infected macrophage cells with various concentrations of Artemisinin (50 µg/mL), Glucantim (400 µg/mL), Artemisinin 50 µg/mL plus Glucantime 400µg/mL and shark cartilage extract after 72 h. The percent of apoptosis (Early and Late Apoptosis) in non-infected macrophage cells induced by 50 μg/mL of Artemisinin, 200 μg/mL of Glucantime, 50 µg/mL of Artemisinin plus 200 µg/mL of Glucantim were 3%, 7.2%, and 4% respectively. Induced apoptosis (Early and Late Apoptosis) in infected macrophage cells after 72 h were 24.5%, 24.5%,42%, and 21% respectively (Figures 8, 9).

## Discussion

Leishmaniasis is one of the 17 most important diseases in the world which WHO insists to eradicate it (1). The disease control relies on removing vectors and reservoirs and providing health care. In the absence of effective vaccines, searching for new, effective, safe and cheap anti- leishmanial compounds were indeed a necessity (2). Plant derivatives have been known to be effective and of broad antimicrobial activity against some of the diseases. One of such plant derivative compounds is Artemisinin. Combination therapy using available drugs aims to reduce cost, toxicity and duration of treatments (5). In this study, we evaluated the possible application of Artemisinin, as an antimalarial drug, in combination with glucantime, as an antileishmanial drug, and shark cartilage as an immunomodulator. Artemisinin is also known to have an antileishmanial effect (3). The effects of Artemisinin on visceral leishmaniasis have widely been studied (18, 36). In Iran, anti-leishmanial activity of Artemisinin on *leishmania infantum* had not been evaluated, so we carried out this first experimental study to examine *in-vitro* effects of Artemisinin alone or combined with shark cartilage extract and Glucantime on *leishmania infantum*.

Certain studies have shown that shark cartilage has some inhibitory effects on angiogenesis, metastasis, cell adhesion, and proteolysis (28). In this study, we examined the effects of shark cartilage on promastigotes and amastigotes of *leishmanaia infantum*. Our results showed that shark cartilage extract alone and Artemisinin alone have an inhibitory effect on growth of promastigotes of *Leishmania infantum*. The inhibitory effects dependent on the dosage and exposure time of drugs, so the growth of the parasite reduces as the doses and exposure time increase. To validate this results, we used repeated measure analysis. We, therefore, found that means of promastigotes growth rates diminished as the drug doses and exposure time increased (*p* < 0.05). The promastigote assay to determine 50% inhibitory concentration (IC50) of the drug showed that the most effective dose of Artemisinin on *L*. *infantum* was 50 μg/mL after 72 h.The combinatorial use of Artemisinin-Shark cartilage extract or Artemisinin-Glucantim on promastigotes of *leishmania infantum* exert more effects than either of combined drugs used alone.These results showed that inhibitory effect of combined drugs is higher than the principal drugs used singly on promastigotes. The count of promastigotes reduced over time when a combination of Artemisinin plus Glucantime or Artemisinin plus shark cartilage extract were applied. Our results also indicated that higher concentrations of drugs produce more significant effects (P < 0.05).

Glucantime alone was highly promastigoticidal only at 400 and 200 µg/mLconcentration (*p* < 0.05), but not at the 100 µg/mL and lower concentrations. In this study, we found significant differences between leishmaniacidal effects of high and low concentration of drugs (*p* < 0.05).

MTT tests indicated that the percentage of parasite surviving after 72 h was also dependent to the concentration of drugs, so that increase in the concentration results in reduction of survival rates (*p *< 0.05). When Artemisinin 100 µg/mL and Glucantime 400 µg/mL were combined, the survival rate of the cells was 65%. Glucantime alone produced a higher survival rate. Showing cytotoxic effect, shark cartilage extract, also, left 73% of parasites alive at 50 µg/mL concentration, but when added to Artemisinin the survival rate was 74%. Artemisinin exerted no toxicity on non-infected macrophages at 3.12-50 µg/mL concentration, but at 100 µg/mL concentration, 79% of the cells were alive. In fact, Artimisinin alone or combined with Glucantime and shark cartilage extract has a little toxic effect on non-infected macrophage cells, but the viability percentage of the infected macrophages at 100 µg/mL and 50 µg/mL concentrations were 20%, and 25% respectively.

Glucantime applied at 100 µg/mL concentration on non-infected and infected macrophage cells produced survival rates of 83% and 20% respectively. When combined Artemisinin and Glucantime were applied at 100 µg/mL concentration, 81% of non-infected macrophage cells and just 31% of infected macrophage cells were alive. Our results also showed that shark cartilage extract alone had no toxic effect on non-infected macrophage cells, but had a slight effect when combined with Artemisinin. The statistical analysis showed a significant correlation between toxicity and both dose or exposure time (*p* = 0.013).

Amastigote assay showed that there is a significant correlation between infectivity of macrophage cells and intracellular amastigotes with drug concentrations, so that the infected cells and the number of amastigotes reduce by increasing drug concentration. This finding confirms the conclusions of previous studies. In 2003, Avery and *et al.* showed that Artemisinin and its analogues are effective on *Leishmania donovani* promastigotes. Therefore, they suggested that Artemisinin analogues may be considered as potential drug candidates against leishmaniasis (37). In 2007, Sen R and *et al*, showed the anti-leishmania activity of Artemisinin on both promastigotes and amastigotes of *Leishmania donovani*, with IC50 values of 160 and 22 µM respectively. They also reported a high safety index (>22-fold) for Artemisinin against macrophage cells (38). In 2010, the same author and co-workers reported antileishmanial activity of Artemisinin on *Leishmania donovani* and *Leishmania infantum* with IC50 values of 100µM to 120µM respectively (39). So, the results of the present study were consistent with other studies.

The flow cytometry of programmed cell death is based on the pro­podium iodide conjugated annexing which bonds to externalized Phosphatidylserine (PS). In alive cells, the phospholipid (PS) is in the inner side of the membrane, while during programmed cell death, this transfers to the external surface of cell membrane, hence its high affinity of annexcin to PS. Flow cytometry analysis indicated that the rate of programmed cell death increased with increasing in amount of drug dose (*p* < 0.05). In the present study, Artemisinin showed apoptotic effect against promastigotes of *L. Infantum. *Previous studies have shown that Artemisinin triggers a programmed cell death or appoptosis. In 2007, Sen R & *et al*. showed that Artemisinin stimulates apoptosis (39). In 2012, Mohammad Islamudin & *et al* proved that Artemisinin induce programmed cell death in *Leishmania donovani* promastigotes (40). 

Cysteine proteases playing a role in metazoan apoptosis have not yet been detected in *Leishmania *parasites, but one metacaspase -like sequence in *L. donovani *was reported (41). Since the survival of the parasite in the vector and in the host macrophages requires strict control of the para­site population, apoptosis may be a useful mechanism to control parasite population and subsequently encourage chemotherapeutic strategies to limit the parasite population (42). In the amastigotes, the entire population expose PS, but this is not necessarily followed by apoptotic death and called apoptotic mimicry (43).

The highest rate of programmed cell death in *L. Infantum* promastigotes induced by Artemisinin was observed at 100 µg/mL concentration, while apoptosis in infected and non-infected macrophage cells were 24% and 2.14%, respectively. So the results of flow cytometry confirmed the results of MTT assay. Our results also showed that combination of drugs induced greater programmed cell death than single drugs. Since *Leishmania *parasites can prevent programmed cell death in infected macrophage cells, Artemisinin alone or in combination with Glucantime and shark cartilage extract may counteract the parasite by inducing programmed cell death in infected macrophages and promastigotes, thus assisting in removal of intracellular parasites. In this study, the results of apoptosis analysis was consistent with other studies using different drugs. Miltefosine can induce apoptosis in *Leishmania donovani* (42). *Alluvium satium* may also induce appoptosis in *Leishmania* genus (44). Drugs such as cantharidin (45), artemether (46), and artemisinin (47) are able to induce apoptosis in *Leishmania major*. Artemisinin has a shorter period of action compared to Glucantim , so the combination may be worth in drug resistance reported from endemic areas in the treatment of visceral leishmaniasis. Also, this study supports the combinatorial use of Artemisinin- Glucantim or Artemisinin- Shark cartilage extract in VL, and intelligibly might be suitable *in-vivo*. These combinations may have concern in Iran, where drug resistance or failure treatment is the serious problem in endemic areas.

## Conclusion

This study suggests that Artemisinin alone or in combination with shark cartilage or Glucantim is effective against the agent of visceral leishmaniasis *in-vitro*.Therefore, Artemisinin plus shark cartilage extract or Glucantim can be further investigated as a medicine for the treatment of visceral leishmaniasis *in*-*vivo*.
